# A novel *cyp26c1*-driven reporter line to study boundaries of retinoic acid signalling during zebrafish development

**DOI:** 10.3389/fcell.2026.1753548

**Published:** 2026-05-25

**Authors:** Rosana Reyes-Pinto, David Arancibia-Altamirano, María J. Vásquez-Ramírez, José Matías Benítez, Aarón Villanueva, Manuela Lahne, Ryan B. MacDonald, Koichi Kawakami, Leonardo E. Valdivia

**Affiliations:** 1 Center for Integrative Biology, Facultad de Ciencias, Ingeniería y Tecnología, Universidad Mayor, Santiago, Chile; 2 Institute of Ophthalmology, University College London, London, United Kingdom; 3 Laboratory of Molecular and Developmental Biology, National Institute of Genetics, Mishima, Japan; 4 Choju Medical Institute, Fukushimura Hospital, Toyohashi, Japan; 5 Escuela de Biotecnología, Facultad de Ciencias, Ingeniería y Tecnología, Universidad Mayor, Santiago, Chile

**Keywords:** CYP26C1, Gal4/UAS, retina, retinoic acid, transgenic, zebrafish

## Abstract

Retinoic acid (RA) signalling is essential for vertebrate development, but the spatiotemporal dynamics of its regulation remain largely uncharacterized in specific cellular contexts. Here, we introduce a novel gene trap transgenic reporter line for the zebrafish *cyp26c1* gene, a key regulator of RA metabolism. Through a screen of a *gal4*-based gene and enhancer trap collection, we isolated the *tg(gSAIGFF104A)* line. In this line, a *gal4* gene trap cassette is inserted into the third intron of the *cyp26c1* gene, allowing it to faithfully drive reporter expression in a pattern that mirrors the endogenous *cyp26c1* expression in zebrafish embryos. Using this reporter, we characterized the expression of *cyp26c1* with cellular resolution, revealing a highly dynamic and localized expression in the developing telencephalon, diencephalon, hindbrain, otic vesicles, pharyngeal arches, and other ectodermal derivatives, as well as in subdomains of the retina. Furthermore, we demonstrate that this line responds to both exogenous and genetic manipulations of RA signalling, particularly within the retina. Our reporter provides a valuable resource for investigating the intricate biology of RA signalling in zebrafish development and disease, offering a tool for tracing and manipulating *cyp26c1*-expressing cells.

## 1 Introduction

Retinoic acid (RA), a bioactive derivative of vitamin A, is an essential signaling molecule that orchestrates a myriad of developmental processes in vertebrates, including cell proliferation, differentiation, patterning and organogenesis (Brown, 2023; Ghyselinck and Duester, 2019; Rhinn and Dollé, 2012; Cunningham and Duester, 2015; Berenguer and Duester, 2022). The functions of RA are mediated by nuclear retinoic acid receptors (RARs), which form heterodimers with retinoid-X receptors (RXRs) to regulate the transcription of target genes by binding to retinoic acid response elements (RAREs) in their promoters (Cunningham and Duester, 2015; Ghyselinck and Duester, 2019; Niederreither and Dollé, 2008). Precise regulation of RA levels is critical for proper embryonic morphogenesis, as its dysregulation acts as a potent teratogen, leading to severe congenital abnormalities (Griffith and Wiley, 1991; Hyatt et al., 1992; Lee et al., 2012; McCaffery et al., 2003; Molineaux et al., 2015; Smith, 2001; Wilson et al., 1953; Zile, 2001). This regulation is achieved through a delicate balance between RA synthesis, primarily by retinaldehyde dehydrogenase (Raldh) enzymes, and RA degradation, mediated by the cytochrome P450 family 26 (Cyp26) enzymes (Pennimpede et al., 2010; Sakai, 2001).

The Cyp26 family plays a central role in establishing and shaping controlled domains of RA activity and responsiveness during vertebrate embryogenesis, (Hernandez, et al., 2007; Pennimpede et al., 2010; Sakai, 2001). To date, three Cyp26 enzymes (Cyp26a1, Cyp26b1, and Cyp26c1) have been identified in human, mice, chick, *Xenopus*, and zebrafish (Kawakami et al., 2005; Lynch et al., 2011; Nelson, 1999; Reijntjes et al., 2004; White et al., 1996). These enzymes are, in turn, responsive to RA, creating a feedback loop in which RA regulates its own availability by controlling *Cyp26* expression (Ross and Zolfaghari, 2011). While the roles of *Cyp26a1* and *Cyp26b1* in establishing and maintaining RA gradients are well-established (Hernandez et al., 2007), the specific physiological function of *Cyp26c1* is not well understood. Importantly, *cyp26c1* exhibits a highly localized and dynamic expression pattern, suggesting a specialized role in fine-tuning RA signalling at specific spatiotemporal boundaries during development (Kawakami et al., 2005; Uehara et al., 2007). This precise expression profile suggests that *cyp26c1*-expressing cells may delineate a sharp RA response, especially within critical territories of the developing central nervous system, such as the forebrain, hindbrain, and retina (Gu et al., 2005; Hernandez et al., 2007). A deeper understanding of the expression and regulation of these molecularly defined cells is important for dissecting the regulatory logic of RA signalling throughout organogenesis.

Despite significant progress, a major challenge in RA research has been the limited availability of tools to visualize and manipulate its dynamic signalling within specific cellular contexts of a developing embryo (Theodosiou et al., 2010; Cunningham and Duester, 2015; Niederreither and Dollé, 2008). Current reporter lines, often based on RAREs, lack cell-type specificity and can show variable expression patterns due to their genomic integration sites (Balkan et al., 1992; Mendelsohn et al., 1991; Reynolds et al., 1991; Li et al., 2011; Qiu et al., 2023; Roberts et al., 2014; Wilson et al., 1990; Kalvaitytė et al., 2024). Furthermore, these reporters are primarily designed to respond to the presence of RA, but they do not necessarily reflect the precise, endogenous boundaries of RA activity. This highlights a need for new, complementary genetic tools that provide a more accurate and dynamic representation of RA-controlled gene expression, particularly those that faithfully replicate the intricate spatiotemporal patterns of less studied regulatory genes like *cyp26c1*. Such tools are essential for studying how RA signalling defines morphological boundaries *in vivo* during development.

Zebrafish (*Danio rerio*) serve as an ideal model organism for developmental studies due to their transparent embryos, rapid development, and genetic tractability (Nüsslein-Volhard and Dahm, 2002). The RA signalling pathways are highly conserved in zebrafish, making them a powerful system for investigating the expression of RA-related genes and the role of RA in vertebrate development and disease (Hawkins and Wingert, 2023). To generate novel transgenic reporter lines two main approaches have been employed: promoter bashing of known RA-regulated genes and forward genetic strategies using enhancer/gene trap screens (Asakawa et al., 2008; Kawakami et al., 2004; Hayashi et al., 2002; Kawakami et al., 2010; Skarnes et al., 2004). Enhancer/gene trap screens, in particular, offer a powerful approach. They involve the random integration of a *gal4* or a fluorescent protein-coding construct into the genome using the Tol2 transposable element, resulting in expression driven by regulatory elements active at that locus (Kawakami et al., 2016; Vásquez-Ramírez et al., 2025). These “trapping” transgenes often faithfully recapitulate the endogenous expression patterns of the targeted genes, providing valuable resources for studying gene function and addressing the limitations of reverse genetic approaches.

In this study, we report the characterization of a novel gene trap zebrafish line, *tg(gSAIGFF104A)*. This line harbours a *gal4* gene trap cassette inserted into the third intron of the endogenous *cyp26c1* gene, enabling it to accurately drive reporter expression that mirrors the endogenous *cyp26c1* pattern. This line enables high resolution imaging and the manipulation of molecularly defined cells in telencephalon, diencephalon, hindbrain, and the retina, offering insights into the development of the central nervous system and other ectodermal derivatives with high spatial and temporal resolution. The *tg(gSAIGFF104A)* line represents an advancement in the zebrafish toolkit available for exploring the intricate biology of RA signalling and its implications for neural development.

## 2 Materials and methods

### 2.1 Zebrafish husbandry

The zebrafish strains used in this study were: AB*, *tg(gSAIGFF104A)* (this study), *tg(5xUAS:EGFP)* (Asakawa and Kawakami, 2009), *tg(UAS:RFP)* ^*yu9Tg*^ (Asakawa et al., 2008), *tg(neuroD:tagRFP)* ^*w69Tg*^ (McGraw et al., 2012), *tg(rx3:Gal4-VP16)* (Weiss et al., 2012) and *tg(atoh7:RFP)* ^*cu2Tg*^ (Zolessi et al., 2006). Embryos were raised at 28.5 °C in E3 medium and staged by hours or days post fertilization (hpf and dpf, respectively) according to Kimmel et al. (1995). For all experiments, we used stable, germline transgenic lines, and embryos harbouring a single insertion of the transgenes, as assessed by inheritance patterns. To reduce embryo pigmentation for imaging, phenylthiourea (PTU) (0.004%; Sigma) was added to the E3 medium (Karlsson et al., 2001).

### 2.2 Screening of transgenic fish

The *tg(gSAIGFF104A)* line is a gene trap line expressing GFF, a modified version of the Gal4 transcriptional activator consisting of the Gal4 DNA-binding domain fused to a tandem repeat of the VP16 transactivation domain (Takeuchi et al., 2015). This line was generated using the *gSAIGFF* gene trap construct, which incorporates a gata6 splice acceptor, the GFF sequence, and the SV40 polyadenylation signal (polyA) to ensure proper termination of the trapped transcript. The integration of this construct was achieved through Tol2 transposon-mediated transgenesis, following established protocols involving the co-injection of the construct with transposase mRNA (Asakawa et al., 2008; Kawakami et al., 2004; Takeuchi et al., 2015).

Our initial screening was performed by analysing fluorescent images of gene trap and enhancer trap lines available in the zTrap database (https://ztrap.nig.ac.jp/ztrap/) (Kawakami et al., 2010) that drive expression in developing eyes. From this dataset, carrier lines were selected for adult crossing. Dechorionated embryos from these crosses were screened and observed daily from 24 to 72 hpf using widefield fluorescence microscopy with an MZ16FA (Leica) dissecting microscope available at the National Institute of Genetics (NIG) in Mishima, Japan. Images were captured using a CCD camera (DFC300 FX, Leica). From these lines, *tg(gSAIGFF104A)* was chosen for further analysis due to its conspicuous transgene expression in a band of cells along the nasal-temporal axis of the retina.

The *tg(gSAIGFF104A)* line was maintained as a stable germline with a single *gal4* integration within the *cyp26c1* locus. For all analyses in this study, embryos from the F5 and F6 generations were used. Inheritance stability and expression fidelity were routinely verified during the selection of new carriers for colony maintenance. Experimental embryos were obtained from double-hemizygous crosses (for *gal4* and UAS-reporter), yielding the expected Mendelian dihybrid phenotypic ratio of 9/16 fluorescent individuals, while outcrosses to wild-type fish yielded the expected 25% fluorescence ratio. The reported expression patterns remained highly consistent across multiple generations, with no detectable mosaicism or variegation.

### 2.3 PCR and sequencing

Genomic DNA (gDNA) from 72 hpf *tg(gSAIGFF104A; UAS:GFP)* GFP-positive larvae was isolated using the HotShot method (Meeker et al., 2007). Briefly, larvae were incubated in 15 µL of alkaline lysis buffer (1X final concentration; NaOH 1.25 M, EDTA 0.01 M, stock 50X) at 95 °C for 30 min. Afterward, 15 µL of neutralization solution (1X final concentration; Tris HCl 2 M, stock 50X) was added. To confirm the insertion of the transgenic line, PCR was performed using gDNA from transgenic larvae with a primer in one of the arms of the *tol2* transposon (T2_For: 5′-ACT​CAA​GTA​AAG​TAA​AAA​AAT​CCC​CAA​AAA-3′) and a primer in the third intron of the *cyp26c1* gene (cyp26c1_Rev: 5′-TGC​AGC​CAA​AAT​CAG​AGA​AGT​TAC-3′), generating a 132 bp product. The PCR protocol is as follows: Initial denaturation at 95 °C for 30 s; Denaturation at 95 °C for 30 s; Annealing at 55 °C for 30 s; Extension at 68 °C for 30 s; 34 cycles from Denaturation to Extension; Final extension at 68 °C for 5 min, and hold at 12 °C. 10X Standard Taq Reaction Buffer (NEB B9004S, New England Biolabs), Taq DNA polymerase (NEB M0267S, New England Biolabs), Forward and reverse primers (10 µM), dNTPs 10 mM mix (dATP, dCTP, dGTP, dTTP; #2840655; Cytiva), template DNA, nuclease free water and DMSO (BM-0660; Winkler) were used for the PCR.

The presence and size of the amplicon were verified by running the PCR product on a 2% agarose gel in 1X TAE buffer. The PCR product was then purified using the GFX PCR DNA and Gel Band Purification Kit (#28903470; Cytiva, United Kingdom) and sequenced using the T2_For primer (Macrogen). The genomic sequence surrounding the insertions was analysed by BLAST search against GRCz11 reference genome in Ensembl.

### 2.4 Microinjection and CRISPR/Cas9

To generate targeted mutations in the *gdf6a* gene, a CRISPR/Cas injection mix was prepared using the AltR S.p. Cas9 Nuclease V3 (Integrated DNA Technologies, IDT: 1081058) and two guide RNAs (gRNAs) designed using CHOPCHOP (Labun et al., 2019). The gRNAs were synthesized using a cloning-free method according to Gagnon et al., 2014. For each gRNA, a 60-base forward oligonucleotide was synthesized, containing a T7 viral promoter for *in vitro* transcription (5′-TAA​TAC​GAC​TCA​CTA​TAG​G-3′), a 20-base spacer region specific to the target site, and an overlap region complementary to the constant oligonucleotide common to all gRNAs. The reverse primer was common for all the gRNAs (Gagnon et al., 2014).

The sequences of the forward oligonucleotides were as follows:

Guide RNA 1:

5′-TAA​TAC​GAC​TCA​CTA​TAG​GCA​ACA​CGG​TAA​ACT​CCA​GGT​TTT​AGA​GCT​AGA​AAT​AGC​AAG-3′

Guide RNA 2:

5′-TAA​TAC​GAC​TCA​CTA​TAG​GTG​CCA​GGA​GCG​CGT​TTG​AGT​TTT​AGA​GCT​AGA​AAT​AGC​AAG-3′

The final concentration of the microinjected solution was 1000 pg/nL of Cas9 and 250 picograms of each gRNA, according to Kroll et al., 2021. The injected volume was 4 nanoliters. Injections were performed at the one-cell stage using a Microinjection Pump (World Precision Instruments, SYS-PV820). Crosses were made between the AB* and *tg(gSAIGFF104A; UAS:GFP)* lines.

### 2.5 Retinoic acid treatment

Stock solutions of all-trans retinoic acid (at-RA, Sigma) were prepared in dimethyl sulfoxide (DMSO; Sigma) and stored at −20 °C until use. Prior to treatment, embryos were manually dechorionated to ensure proper exposure to the compound. RA was supplied in E3 media at a final concentration of 100 μM, with 2% DMSO as a vehicle control. Embryos were treated with RA from 28 hpf until 52 hpf, a critical window for transgenic expression, and processed for imaging.

### 2.6 Sample processing for imaging

Embryos at 24, 48, 52, and 72-hpf were anesthetized with 0.4% tricaine (MS-222, Sigma) and transferred to 4% paraformaldehyde (PFA) in phosphate-buffered saline (PBS) for overnight fixation at 4 °C. Whole embryos or dissected brains and eyes were counterstained with TO-PRO-3 (Invitrogen T3605) and reserved for further processing and imaging modalities. Cleared, RA-treated, and HCR samples were embedded in 1% low-melting-point agarose (LMP agarose, Sigma) in PBS, and mounted for confocal and light sheet imaging. Larval brain dissection was performed according to Turner et al. (2014).

### 2.7 Tissue clearing

Embryos, dissected brains and eyes were cleared using CUBIC (Susaki et al., 2014; Pende et al., 2020), with modifications. CUBIC-1.1 solution consists of 10% (v/v) N, N, N′, N′-Tetrakis(2-Hydroxypropyl) ethylenediamine (Sigma-Aldrich, 122262), 5% (v/v) Triton X-100 and 5% (m/v) urea (Sigma-Aldrich, U5128). CUBIC-2 solution consists of 10% (m/v) 2,2′,2″-Nitrilotriethanol, Tris(2-hydroxyethyl) amine (Sigma-Aldrich, 90279), 50% (m/v) sucrose (Sigma-Aldrich, S0389) and 25% (m/v) urea. Tissues were cleared in CUBIC-1.1 solution at 37 °C until transparency was reached and imaged using confocal microscopy or transferred and equilibrated in CUBIC-2 solution for refractive index (RI) matching at 1.45 for light sheet microscopy.

### 2.8 Hybridization Chain Reaction (HCR)

72-hpf embryos were fixed in 4% PFA overnight at 4 °C and dehydrated in a methanol gradient for permeabilization and long-term storage at −20 °C if required. Embryos were gradually rehydrated to PBSTr (PBS with 0.05% Triton X-100), permeabilized by Proteinase K treatment (10 ng/mL in 0.05% PBSTr) and post-fixed in 4% PFA for 20 min at room temperature. HCR protocol was performed as described by the manufacturer (Molecular Instruments). Custom HCR 3.0 probes for *cyp26c1* mRNA (Supplementary Table S1) were designed with a published script (Kuehn et al., 2021) using the coding sequence of *cyp26c1* and aligned against the transcriptome to exclude off-target detection.

### 2.9 Confocal imaging

Imaging was performed on a Zeiss LSM 710 confocal microscope using the HeNe633, HeNe543 and 458/488/514 multi-line Argon lasers. 16-bit images were acquired at a pinhole size of 1 airy unit and employing the Plan-Apochromat 10x/0.3 M27 and EC Plan-Neofluar 20x/0.50 M27 objectives. Samples were imaged in 1% LMP agarose within custom-made glass-bottom chambers. For cleared samples, the imaging chamber was filled with CUBIC-1.1 solution for refraction index matching at 1.38.

### 2.10 Light sheet imaging

Imaging of fixed and live samples was performed in a Zeiss Light Sheet 7 microscope with 405/488/561/638 nm laser lines, using glass capillaries for mounting. For fixed material, cleared samples were RI matched at 1.45 in CUBIC-2 solution. Images were acquired with a Clr Plan-Neofluar 20x detection objective (1.0 Corr, nd = 1.45) and two LSFM 10×/0.2 illumination objectives. Image acquisition utilized dual-side illumination with pivot scan, a bit depth of 16-bit and a light sheet thickness of 2 µm.

For live imaging, embryos were maintained in a chamber with E3 medium heated to 28.5 °C. *tg (gSAIGFF104A; UAS:GFP)* embryos were anaesthetized with 0.04% tricaine and mounted in 1% low-melting-point agarose (Sigma) in E3 medium with PTU. Time-lapse z-stacks were collected from a lateral view at 3-min intervals for 12 h, beginning at 54 hpf. Images were acquired by dual-side illumination with pivot scan, a bit depth of 16-bit, and a light sheet thickness of 2 µm.

### 2.11 Image analysis

Fiji (Schindelin et al., 2012) was employed for general image processing. Particle tracking analysis on maximal intensity projection (MIP) time lapse image sequences was performed using the Mastodon plugin, which is optimized for cell tracking in large datasets and derived from the TrackMate plugin (Tinevez et al., 2017). Dual-side fusion, color-coded projection, and light sheet processing were conducted using Zen Blue 3.4 (Zeiss). Imaris 9 (Oxford Instruments) was utilized for three-dimensional (3D) rendering and surface segmentation.

## 3 Results

### 3.1 The *tg(gSAIGFF104A)* transgene drives GFP expression in the developing nervous system and other ectoderm derived tissues

**FIGURE 1:**
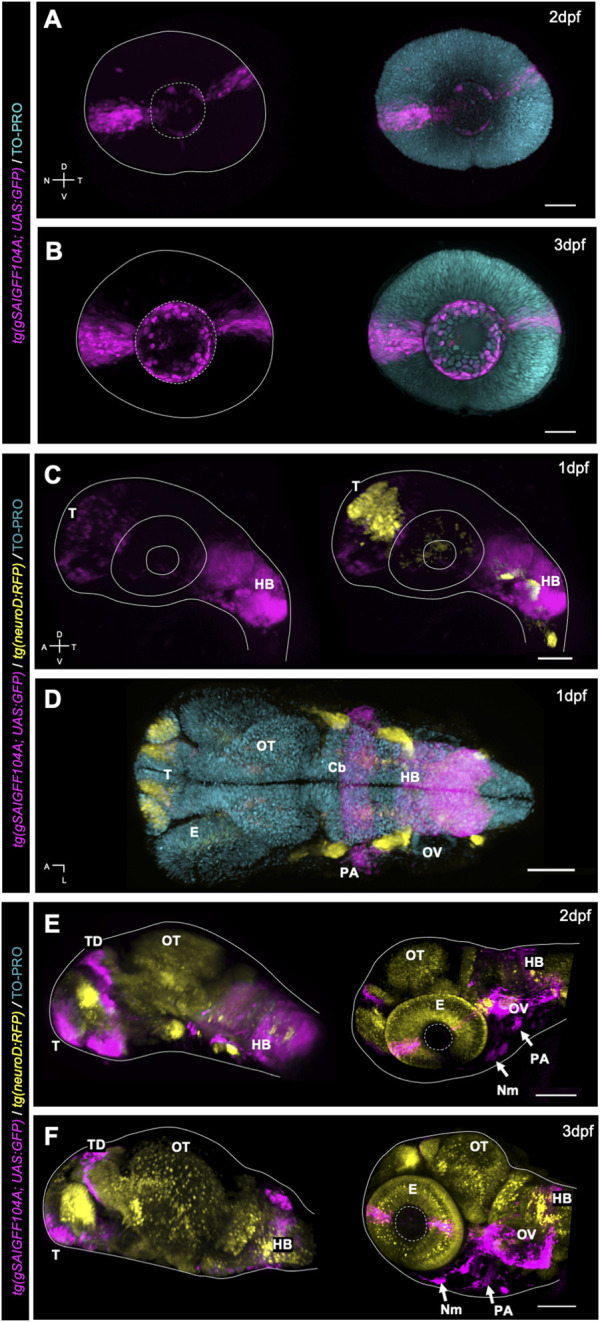
*tg(gSAIGFF104A)* drives expression in a stripe along the nasal-temporal axis of the developing zebrafish retina. **(A**,**B)** show 3D reconstructed lateral views of *tg(gSAIGFF104A; UAS:GFP)* transgenic whole eyes at 2 dpf and 3 dpf, respectively. The GFP reporter expression (magenta) is observed in a highly specific stripe along the nasal-temporal axis of the retina. Sparse GFP-positive cells are also visible within the lens (indicated by dashed circles). Nuclei were visualized using TO-PRO-3 counterstaining (cyan). Scale bar: 50um. **(C–F)** 3D reconstructions of whole-mounts and dissected brains showing the expression dynamics of the *tg(gSAIGFF104A; UAS:GFP)* reporter line (magenta) from 1 to 3 dpf. Transgenic animals were outcrossed with the *tg(neuroD:tagRFP)* line (yellow) for enhanced tissue context. C and D show transgenic embryo views at 1 dpf. Images C, E, and F present a dissected brain (left) alongside a mounted head (right) to provide full spatial context. E and F show lateral views of embryos at 2 dpf and 3 dpf, respectively. Notable expression is observed in the rhombomeres/hindbrain (HB), Cerebellum (Cb), boundary between telencephalon and diencephalon (TD), telencephalon (T), eye **(E)**, and otic vesicle (OV). Additional expression is seen in the pharyngeal arches (PA) and cranial neuromasts (Nm) (indicated by arrows in E and F). Additional abbreviation: OT, optic tectum. Scale bar: 100um.

To identify transgenic lines that drive gene expression in developing zebrafish eyes, we initially screened incrosses of 71 lines from a pool of 255 candidates. These lines were preselected based on their preliminary expression patterns in the developing brain and eye after being crossed with a *tg(UAS:GFP)* responsive fish. From this screening, we isolated a novel transgenic line, *tg(gSAIGFF104A)*, which exhibits a striking and highly specific expression pattern in the retina. This line displayed a distinct band of GFP-positive cells along the nasal-temporal axis of the developing retina at 2 dpf (Figure 1A). By 3 dpf, this well-defined band of GFP-positive cells was readily detectable, showing a broader distribution within the nasal retina (Figure 1B). At both 2 and 3 dpf, a sparse number of cells were also labelled in the lens. The highly specific retinal expression pattern reflects targeted activation of the reporter transgene within a retinal subdomain, underscoring its value as a tool for investigating retinal development and the signalling pathways that define specific cell populations.

In addition to the expression in the retina, significant *tg*(*gSAIGFF104A)*-driven expression was observed in various other embryonic regions. Specifically, we detected strong GFP fluorescence in the telencephalon, hindbrain, pharyngeal arches, and anterior lateral line (Figures 1C–F). At 24 hpf, GFP expression in the hindbrain closely aligns with developmentally regulated compartmentalization events (Figures 1C,D), serving as a reliable marker for delineating tissue boundaries crucial for proper morphogenesis. At this stage, expression was also prominent in part of the otic vesicle (Figure 1D). As development progressed, expression in the hindbrain, otic vesicle and pharyngeal arches remained prominent, with additional labelling detected in the boundary between telencephalon and diencephalon, and the anterior lateral line (Figures 1E,F), highlighting the progressive activation of expression in both central and peripheral nervous domains.

The observed GFP expression pattern in the developing brain partially overlaps with the neuronal marker *neuroD*, as demonstrated by outcrossing the *tg(gSAIGFF104A; UAS:GFP)* line with the *tg(neuroD:tagRFP)* reporter line (Figures 1C,F). These results are consistent with the idea that RA activity intersects with regions of early neuronal differentiation. Overall, our data demonstrates that the *tg*(*gSAIGFF104A; UAS:GFP)* transgenic line marks relevant nervous system subdomains, sensory organs, and other ectoderm-derived tissues throughout early development. This precise and dynamic expression pattern makes it a valuable tool for delineating tissue boundaries crucial for morphogenesis.

### 3.2 The expression of the *tg(gSAIGFF104A)* transgenic line is driven by the *cyp26c1* locus

**FIGURE 2:**
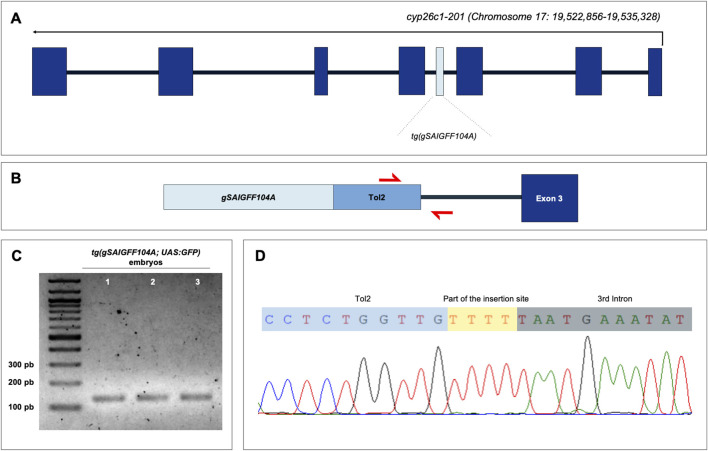
Genomic insertion site of the *tg(gSAIGFF104A)* transgene within the *cyp26c1* locus. **(A)** Schematic representation of the *cyp26c1* locus, showing the insertion site of the *Tol2* element within the third intron of the gene, in the negative strand of chromosome 17. **(B)** Detailed representation of the specific genomic context at the transgenic line insertion. Red arrows indicate the primers designed and used for the PCR-based confirmation of the insertion site. **(C)** Gel electrophoresis results of PCR products used to identify the insertion site in three different transgenic embryos from an incross; all three samples successfully generated the expected 132 base pair product, confirming the presence of the insertion. **(D)** Sequence chromatogram of the region amplified by PCR, confirming the precise location of the *Tol2* insertion. The sequence colours denote: Blue represents one arm of the *Tol2* element (part of the transgenic line); Yellow highlights the insertion junction/site; and Grey indicates the adjacent region of the *cyp26c1* intron.

Given the transgenic pattern observed, we aimed to identify the gene responsible for driving this expression. According to the zTRAP database, the *tg(gSAIGFF104A)* construct was inserted within the third intron of the *cyp26c1* gene, which encodes a 7-exon *retinoid 4-hydroxylase* responsible for degrading RA, thereby restricting its signalling and controlling its concentration levels (Figure 2A) (Zhong et al., 2018). To validate the transposon insertion, we performed a genotyping PCR using primers located in the *Tol2* sequence of the transgene and the adjacent genomic region of *cyp26c1* (Figure 2B). The amplification and sequencing of a 132 bp product confirmed that the *tg(gSAIGFF104A)* transgene is correctly inserted 326 bp downstream of the start of the third intron of *cyp26c1*, thereby creating a gene trap (Figures 2C,D).

**FIGURE 3:**
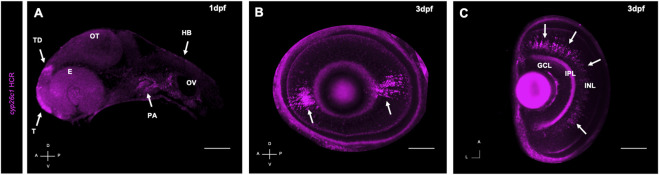
Hybridization Chain Reaction (HCR) confirms *cyp26c1* expression aligns with GFP in *tg (gSAIGFF104A; UAS:GFP)* embryos. **(A)** Lateral view of a 3D reconstruction of a hemisphere showing HCR detection of *cyp26c1* mRNA (magenta) at 1 dpf highlights expression at the telencephalon-diencephalon (TD) boundary, telencephalon (T), the eye (E), the otic vesicle (OV) and pharyngeal arches (PA). 3D views derived from lateral and dorsal eye substacks shown in **(B,C)** highlight the distribution of *cyp26c1* mRNA within defined retinal subregions at 3 dpf (white arrows in B and C). Abbreviations: OT, optic tectum; GCL, ganglion cell layer; IPL, inner plexiform layer; INL, inner nuclear layer; boundary between telencephalon and diencephalon (TD), telencephalon (T), eye (E), otic vesicle (OV), anterior (A), posterior (P), dorsal (D), ventral (V), and lateral (L). Scale bar = **(A)** 100um; **(B,C)** 50um.

The insertion of the *tg*(*gSAIGFF104A)* transgene within the third intron of the *cyp26c1* gene supports the idea that the *gal4*-encoding sequence is actively transcribed and expressed alongside *cyp26c1*. Consistently, we compared the retinal GFP expression pattern with the spatial distribution of *cyp26c1* mRNA using HCR. The two expression patterns showed close correspondence (Figures 3A–C), reinforcing the reliability of the *tg(gSAIGFF104A)* line as a driver that recapitulates the expression of *cyp26c1*.

### 3.3 The *tg(gSAIGFF104A)* transgene drives gene expression in both neuronal and non-neuronal cell types during retinal development

**FIGURE 4:**
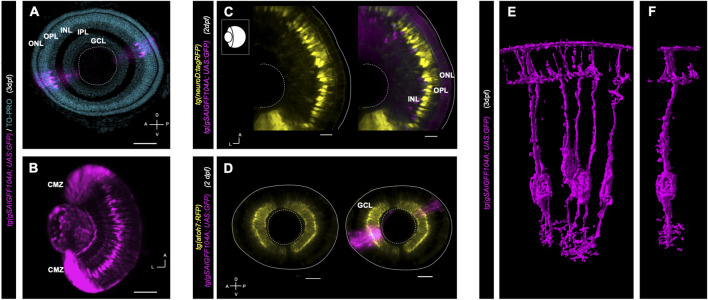
The *tg (gSAIGFF104A)* drives gene expression in neuronal and non-neuronal cell types during retinal development. **(A)** An optical section through a *tg (gSAIGFF104A; UAS:GFP)* eye at 3 dpf reveals GFP expression across the layers of the central retina (magenta), with a conspicuous expression pattern in the Inner Nuclear Layer (INL). **(B)** A dorsal view of a 3D reconstruction of the tg *(gSAIGFF104A; UAS:GFP)* eye at 3 dpf shows that the GFP localization and expression pattern delineate Müller cells and the CMZ within the expression band (magenta). Note the GFP-positive stripe extending from the CMZ, to the central retina. **(C)** Substack optical section through the retina at 2 dpf shows co-localization of GFP (magenta) with *tg (neuroD:tagRFP)* expression (yellow), indicating labeling in neuronal cell types (bipolar and amacrine cells). **(D)** Lateral view of an optical section of the retina at 2 dpf showing cells co-labelled with GFP (magenta) and the *tg (atoh7:GFP)* transgene (yellow), confirming expression in a subset of retinal ganglion cells. **(E–F)** Segmentation and surface rendering reveal strikingly clear labelling of Müller glia in *tg(gSAIGFF104A)* eyes, enabling visualization of the anatomical details of both small clusters **(E)** and individual GFP-positive Müller cells (magenta) **(F)**. Abbreviations: INL, inner nuclear layer; CMZ, ciliary marginal zone; A, anterior; P, posterior; D, dorsal; V, ventral; L, lateral. Scale bar = **(A,B)** 50um; **(C)** 20um and **(D)** 40um.

At 72 hpf, the zebrafish retina is laminated. At this stage, the *tg(gSAIGFF104A)* line drives gene expression in a striking and spatially refined pattern within the medial region of the developing retina (Figure 1). Using high-magnification captures of the retina we found UAS-driven GFP expression across the ganglion cell layer (GCL), inner nuclear layer (INL), and outer nuclear layer (ONL) (Figure 4A). Optical sections of *tg(gSAIGFF104A; UAS:GFP)* along the dorsal ventral axis of the retina reveal trails of cells emerging from the ciliary marginal zone (CMZ), a reservoir of multipotent retinal progenitors in fish (Figure 4B) (Raymond et al., 2006; Centanin et al., 2011).

To confirm expression in different retinal cell types, we conducted co-labelling experiments by outcrossing our driver line into established transgenic markers for progenitors and differentiated cells in the retina. At 48 hpf, GFP expression was found in cells that also expressed the neuronal marker *tg(neuroD:tagRFP),* as well as in cells expressing *tg(atoh7:mRFP)* that define committed progenitors and subsets of amacrine and bipolars cells, as well as retinal ganglion cells, respectively (Figures 4C,D) (Zolessi et al., 2006; Raymond et al., 2006). Notably, *tg(gSAIGFF104A; UAS:GFP)* line selectively labels a distinct subset of Müller glia, the last lineage to be born in the embryonic zebrafish retina (Furukawa et al., 2000), which are prominently marked by GFP (Figures 4E,F). This widespread expression pattern in the retina highlights the utility of the *tg(gSAIGFF104A)* line to mark both neuronal and non-neuronal cell types as the tissue matures. Together, our co-labelling results and detailed morphological description validate *tg(gSAIGFF104A)* as a robust driver line that faithfully labels retinal cells, from progenitor to differentiated neurons and glia, during embryonic and larval retinal development.

### 3.4 Versatility of the *tg(gSAIGFF104A)* transgenic line

**FIGURE 5:**
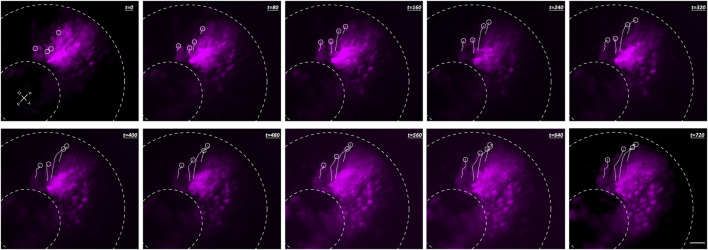
Time-lapse imaging of *tg (gSAIGFF104A; UAS:GFP)* showing the dynamic contribution of labelled CMZ progenitors to the central retina. Image stacks 171 um of 0.42 um optical sections were captured every 3 min for a total duration of 12 h, commencing at 54 hpf, which is defined as t = 0. Individual cells are marked with circles, and their trajectories are shown as lines, indicating the migratory path of labelled CMZ progenitors. The time “t” is indicated in minutes. Anterior is oriented toward the upper right. The large, dashed line marks the border of the eye, and the small, dashed line indicates the border of the lens. GFP is in magenta colour. Abbreviations: A, anterior; P, posterior; D, dorsal; V, ventral; L, lateral Scale bar = 20 um. See also Supplementary Movie S1.

The pattern of labelled cells across all three retinal lineages in *tg(gSAIGFF104A; UAS:GFP)* is reminiscent of the ARCoS that arise from the CMZ, as described by Centanin et al., 2011. To gain deeper insights into the behaviour of the CMZ retinal progenitors labelled by the *tg(gSAIGFF104A; UAS:GFP)* line, we conducted time-lapse imaging from 54 to 66 hpf, a period when the CMZ is active (Cerveny et al., 2010; Valdivia et al., 2016). This approach revealed that GFP-labelled cells derived from the CMZ actively migrate and integrate within the central retina, demonstrating the dynamic nature of retinal growth and the contribution of CMZ progenitors to retinal expansion and architectural maintenance (Figure 5; Supplementary Movie S1). Collectively, these findings establish the *tg(gSAIGFF104A)* driver as a powerful tool for studying the spatial and temporal dynamics of post-embryonic retinal progenitors *in vivo*.

**FIGURE 6:**
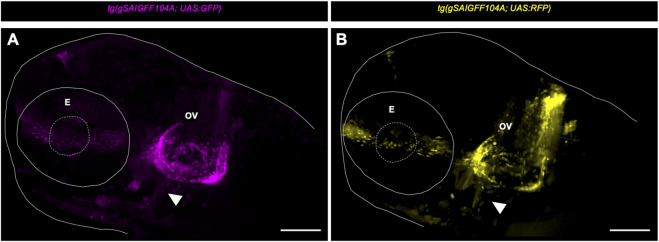
The *tg (gSAIGFF104A)* line enables versatile expression of UAS-based reporters. **(A)** A maximum projection image of a 3 dpf *tg (gSAIGFF104A)* embryo shows GFP expression in the eye (E), otic vesicle (OV), and pharyngeal arches (indicated by arrowheads; GFP in magenta). **(B)** When crossed to the *tg(UAS:RFP)* reporter line, the resulting double-transgenic embryos replicate the same spatial pattern with RFP (yellow). Scale bar = 100um.

Finally, to illustrate additional versatility of the *tg(gSAIGFF104A)* line, we outcrossed it with the *tg(rx3:Gal4-VP16); tg(UAS:RFP)* (Weiss et al., 2012) reporter line. This crossing enabled visualization of *tg(gSAIGFF104A)*-driven expression using an alternative fluorescent marker. Consistent with the previous observations, RFP expression closely mirrored the GFP pattern confirming the ability of the *tg(gSAIGFF104A)* line to drive expression of other UAS-based transgenes. Importantly, we detected no evidence of variegation or ectopic expression (Figure 6).

### 3.5 The *tg(gSAIGFF104A)* transgenic line shows tissue-specific response to retinoic acid signalling

**FIGURE 7:**
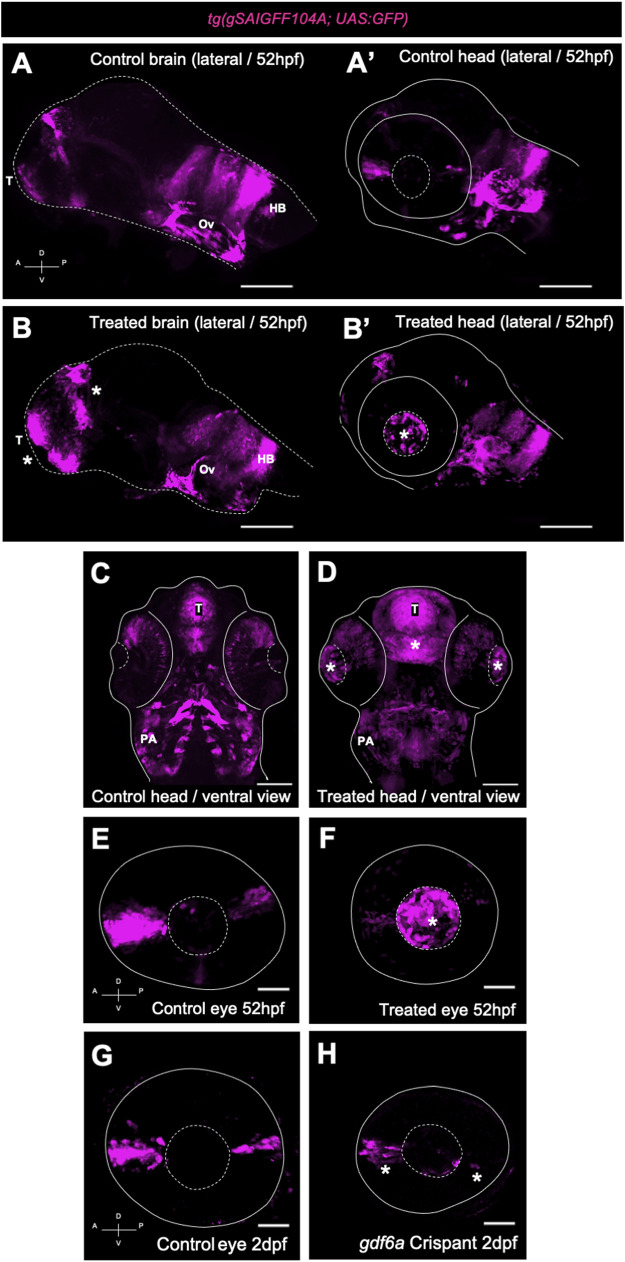
The *tg (gSAIGFF104A)* line responds to retinoic acid signalling perturbations and loss of *gdf6a* function. All images are 3D reconstructions of whole brains and eyes. Images **(A,A′,C)** serve as vehicle controls (DMSO) for RA-treated samples in **(B,B′,D)**. Treated embryos were incubated with 100uM RA from 28 to 52 hpf. **(A,B)** present lateral views of dissected brains, illustrating the overall effect of RA-induced signalling on the transgenic expression pattern **(B)**, asterisks). **(A′,B′)** display whole-mount heads, highlighting an expansion of the GFP signal (magenta) following RA treatment in **(B′)**, asterisk) compared to the control **(A′)**. **(C,D)** provide ventral views of control and RA-treated transgenic larvae, further emphasizing the ventral upregulation of GFP expression in the RA-treated condition **(D)**, asterisk). **(E,F)** present isolated eyes, offering a clearer visualization of RA-induced changes in GFP expression, with the treated eye **(F)**, exhibiting a marked increase relative to the control **(E)** asterisk). **(G,H)** compare the baseline transgenic expression pattern **(G)** with that of a *gdf6a* loss-of-function embryo **(H)**, revealing an overall reduction of expression in the retina (asterisks in **(G)**. Abbreviations: OV, otic vesicle; HB, hindbrain; T, telencephalon; PA, pharyngeal arches; anterior, A; posterior, P; dorsal, D; ventral, V; lateral, L. Scale bar = **(A–D)** 100um; **(E–H)** 50um.

RA signalling plays a critical role in dorsoventral retinal patterning, with components of RA synthesis expressed in a polarized manner in the retina (Molotkov et al., 2006). Furthermore, RA has been shown to induce the expression of *cyp26* enzyme-coding genes, which degrade RA and thus regulate its own levels (Molotkov et al., 2006; Hu et al., 2008; Thatcher and Isoherranen, 2009; Lupo et al., 2011). Given the expression of the *tg(gSAIGFF104A; UAS:GFP)* reporter in the medial region of the developing eye, we hypothesized that modulating RA levels could influence the transgene expression. To test this, we first treated *tg(gSAIGFF104A; UAS:GFP)* heterozygous embryos with exogenous RA from 28 hpf to 52 hpf, a timing where the *cyp26c1* reporter is expressed in the retina. We used heterozygous embryos to control for transgene copy number. Consistent with the known role of RA, acute treatment with a 100 µM RA concentration resulted in craniofacial malformations (Figure 7) (Berenguer et al., 2018). Notably, together with a small eye phenotype, the reporter expression is downregulated in the retina but increased within the developing lens (Figures 7E,F). This behaviour is consistent with the tissue specific regulation previously described for *cyp26c1*, which can be either upregulated or downregulated depending on the cellular context (Pennimpede et al., 2010; Reijntjes et al., 2004; Reijntjes et al., 2005; Luu et al., 2001; White et al., 2007). These findings indicate that, to some extent, the *tg(gSAIGFF104A)* line responds to RA, highlighting its utility for studying RA signalling dynamics in aspects of eye development.

To further explore this idea, we analyzed the loss-of-function of the *gdf6a* gene, which is known to counteract ventrally produced RA and arrest the growth of the eye (French et al., 2009; Valdivia et al., 2016). We found that somatic mutagenesis of the *gdf6a* locus using CRISPR/Cas results in smaller eyes, together with a reduction in the transgenic reporter signal, suggesting that increased levels of RA signalling accompany this phenotype (Figures 7D–H).

## 4 Discussion

In this study, we introduce *tg(gSAIGFF104A)*, a novel zebrafish gene trap transgenic line. The line features a *gal4* insertion within the *cyp26c1* locus, which encodes a key regulator of RA metabolism. Through its combination with *tg(UAS:GFP)*, this line drives GFP expression within specific subdomains of the developing nervous system, including the retina, as well as in other ectoderm-derived tissues. This versatile tool is particularly well suited for studies aiming to delineate tissue boundaries and track cell behaviour during early embryonic development.

The utility of the *tg(gSAIGFF104A)* line hinges fundamentally on its structure and fidelity. The line was generated via a Tol2-mediated gene trap insertion localized within the third intron of the *cyp26c1* locus. The construct architecture, which integrates both splicing acceptor and donor sites, suggests that *gal4* transcription proceeds through an internal fusion event with the endogenous transcript (Davison et al., 2007). While this insertion holds the potential to disrupt gene function, the assessment of a direct mutagenic effect falls outside the scope of this work; we have not detected an obvious morphological phenotype in incrosses of the line (data not shown). Crucially, the observed GFP reporter expression (Figure 1) closely mirrors the endogenous expression domains of *cyp26c1* (Gu et al., 2005; Balciuniene et al., 2013). This robust correspondence validates the transgene as a precise reporter of *cyp26c1* transcriptional activity.

The transgenic line reveals several biologically relevant features. The conspicuous hindbrain labelling at 24 hpf is particularly notable, given its consistency with the established role for RA signalling during hindbrain compartmentalization and rhombomere formation (White et al., 2007; Hernandez et al., 2007; Addison et al., 2018). Additional expression in otic vesicles and pharyngeal arches points to a potential involvement in the development of early ectodermal derivatives (Maier and Whitfield, 2014; Mackowetzky et al., 2022; Isabella et al., 2020). The partial overlap with the neuronal marker *neuroD* in *tg(neuroD:tagRFP)* embryos further suggests that *cyp26c1* may influence specific neuronal populations, underscoring the complexity of RA signalling in the developing nervous system (Linville et al., 2004). These observations indicate the substantial utility of our line in revealing fine morphological and molecular events during neuronal differentiation, particularly when combining the line with other relevant reporters.

A remarkable strength of *the tg(gSAIGFF104A)* lies on its ability to drive expression in retinal progenitor cells, neurons and Müller glia while confining this expression to a distinct horizontal stripe within the zebrafish retina. Through co-labelling experiments with the transgenic markers *tg(neuroD:tagRFP)* and *tg(atoh7:RFP)*, 3D reconstruction, and assessment based on retinal layer position, we confirmed that the *tg(gSAIGFF104A; UAS:GFP)* broadly labels diverse retinal cell types (Kay et al., 2001; Poggi et al., 2005; MacDonald et al., 2015). Indeed, high-magnification imaging at 72 hpf revealed GFP expression across all retinal layers, demonstrating the utility of the line for studying the dynamic contributions of retinal progenitors to retinal architecture. Furthermore, time-lapse imaging from 54 to 66 hpf showed that GFP-positive cells originating in the CMZ actively contribute to the central retina, highlighting the critical role of CMZ progenitors in retinal growth and maintenance (Centanin et al., 2014). This restricted expression within the CMZ subdomain along the dorsal-ventral axis is a powerful asset, as it allows for precise manipulation of gene expression within the stem cell niche. Combining the precise labelling of *tg(gSAIGFF104A)* with blastula cell transplantations will constitute a robust tool for single cell lineage tracing and high-resolution analysis.

The *tg(gSAIGFF104A)* line exhibits a tissue-specific response to RA signalling, a critical regulator of dorsoventral retinal patterning in vertebrates (Wagner et al., 2000; Sen, 2005; Molotkov et al., 2006). Our results show that loss-of-function of *gdf6a*, which normally counteracts ventrally produced RA, resulted in smaller eyes and reduced GFP expression in the retina. This observed phenotype is likely due to an increase in the expression of genes involved in RA synthesis, as *gdf6a* loss-of-function is known to elevate RA signalling in developing zebrafish eyes (Asai-Coakwell et al., 2007; Valdivia et al., 2016). However, we cannot entirely exclude the possibility that primary patterning defects also contribute to the observed phenotype. Consistent with the established role of RA in modulating *cyp26c1* expression, exogenous RA treatment led to classic craniofacial malformations and a downregulation of GFP in the retina, while upregulating it in the lens (Figure 7). Taken together, both genetic manipulation of RA levels and exogenous RA treatment support the idea that increased RA levels can modify *cyp26c1* expression or the formation of cells that express it. This strongly underscores the utility of the *tg(gSAIGFF104A)* line for investigating RA signalling dynamics, particularly the function and regulation of the poorly characterized *cyp26c1* enzyme and its role in eye development (Marsh-Armstrong et al., 1994).

Another significant advantage of the *tg(gSAIGFF104A)* line is its combinatorial utility within the Gal4-UAS system, allowing for versatile experimental applications. When crossed with *tg(UAS:RFP)*, the driver line exhibited the same precise expression pattern as observed with *tg(UAS:GFP)*, with no evidence of variegation or ectopic expression (Halpern et al., 2008). This demonstrated tunability confirms that the line provides robust, interchangeable expression control over any UAS-driven reporter, serving as a powerful and flexible tool for studying *cyp26c1*-mediated developmental processes and probing the dynamics of various gene expression cascades.

It is worth noting that another *cyp26c1* gene trap line has been previously reported, featuring a *gal4* insertion in an exon, which likely disrupts the function of the endogenous gene (Balciuniene et al., 2013). While that line could serve as a loss-of-function allele, its phenotypic consequences have not yet been analyzed. Despite this, the expression pattern of the published line is consistent with our *tg(gSAIGFF104A)* line, suggesting that both lines capture the endogenous *cyp26c1* expression profile (Balciuniene et al., 2013).

The *tg(gSAIGFF104A)* line represents a significant resource that opens exciting avenues for future research. One particularly promising direction is to further investigate the behaviour of a subset of CMZ progenitors and their contribution to the central retina, analogous to the ARCoS described by Centanin et al. (2014). This approach could yield insights into the cellular and molecular mechanisms underlying retinal growth. Additionally, it is noteworthy that the expression pattern observed in zebrafish appears to be conserved across vertebrates. In both mouse (Wagner et al., 2000; Sakai et al., 2004; Dräger and Olsen, 1981) and chick (da Silva and Cepko, 2017), a conspicuous tripartite retinal compartmentalization has been described: a ventral RA-rich zone, a dorsal RA-poor zone and a horizontal RA-poor strip (Wagner et al., 2000); this central strip is marked by expression of *Cyp26c1* and *Cyp26a1*, where the latter is also detected in the developing fovea of rhesus monkeys (Krueger et al., 2023) and human, particularly in Müller glia (Cowan et al., 2020; Krueger et al., 2023). These findings suggest a conserved role for Cyp enzymes to establish and delineate critical boundaries during retinal patterning across species, potentially influencing photoreceptor/RGC production or topographic map formation. Therefore, the *tg(gSAIGFF104A)* line may help to unveil previously unrecognized structural or functional features of this retinal region in zebrafish. Finally, the *tg(gSAIGFF104A)* line holds substantial utility for exploring the role of *cyp26c1* in other developmental contexts, such as craniofacial development, neurogenesis, and hindbrain compartmentalization, where RA signalling is known to relevant (Niederreither and Dollé, 2008; Janesick et al., 2015; Dubey et al., 2018; Frank and Sela-Donenfeld, 2018). The ability to cross the *tg(gSAIGFF104A)* line with other UAS-based responsive lines, such as optogenetic tools or biosensors, provides a direct means to enable sophisticated functional studies of *cyp26c1*-expressing cells *in vivo.*

In summary, *the tg(gSAIGFF104A)* transgenic line provides a novel and versatile resource for studying retinal development, RA signalling, and the behaviour of retinal progenitors *in vivo*. Its ability to faithfully recapitulate *cyp26c1* expression, label diverse cell types, and drive expression of other UAS-based constructs makes it a valuable addition to the zebrafish toolkit. Future studies using this line will be instrumental in illuminating the molecular mechanisms underlying retinal progenitor behaviour, deepening our understanding of the role of RA signalling in eye and retinal development, and uncovering broader functions of *cyp26c1* in vertebrate embryogenesis and disease.

## Data Availability

The original contributions presented in the study are included in the article/Supplementary Material, further inquiries can be directed to the corresponding author.
