# Supplementary material for: A novel cyp26c1-driven reporter line to study boundaries of retinoic acid signalling during zebrafish development
Source: Front Cell Dev Biol. 2026 May 25;14:1753548. doi: 10.3389/fcell.2026.1753548 (PMC13247542; doi:10.3389/fcell.2026.1753548)
Supplement: Supplementary file 1 [file Table1.docx]

**Supplementary table 1. List of HCR probes for *cyp26c1* gene.**

| *Cyp26c1* | Amplifier | H1 | H2 |
| --- | --- | --- | --- |
|  | B1 | GAGGAGGGCAGCAAACGGAACCCGGGTGATGCTCCACCTGAACTC | GCGGGAGTGGGAGTTTACACGTCCTTAGAAGAGTCTTCCTTTACG |
|  |  | GAGGAGGGCAGCAAACGGAACTGCACCGGTCACTCGGATGAGAGG | CACCCAGCAGAATCTTCCGGATGTTTAGAAGAGTCTTCCTTTACG |
|  |  | GAGGAGGGCAGCAAACGGAATGCTCTGGGGCCACTGTGTGCAGAC | GAGTGTTGGGTCCCAGGATGATTCGTAGAAGAGTCTTCCTTTACG |
|  |  | GAGGAGGGCAGCAAACGGAATCCACAGACCCGGTCTCAGTGCACC | GTGAGTGACTTGGCAGCGGTGTAAATAGAAGAGTCTTCCTTTACG |
|  |  | GAGGAGGGCAGCAAACGGAATTCCCTTGCGCAGGCCGCTAACAGG | CAGAGTGCAGAATCTCACGAGCTCTTAGAAGAGTCTTCCTTTACG |
|  |  | GAGGAGGGCAGCAAACGGAAGCTGGCCGTCGTCGAGTGGGCAGCG | CAGCTGCATGATCAGGGATGTTGAATAGAAGAGTCTTCCTTTACG |
|  |  | GAGGAGGGCAGCAAACGGAAGCTCTGGCTCGTTCGCTCACGTCTG | GTGATCAAGCCCTCGCTCTCCAGCTTAGAAGAGTCTTCCTTTACG |
|  |  | GAGGAGGGCAGCAAACGGAATCCAGCCCTTCGGGATCTGATAACC | GTGTGTCGCGGATGCTGTACATGACTAGAAGAGTCTTCCTTTACG |
|  |  | GAGGAGGGCAGCAAACGGAAAAAGGCACGTAACTAAAACGCTCGC | CCGATGCATCTCCTCACACCGCCTCTAGAAGAGTCTTCCTTTACG |
|  |  | GAGGAGGGCAGCAAACGGAACCGCTGTGGCCAGTAGCTCCACTGC | GATATGTTTGGGTTGCCAGAGTGCATAGAAGAGTCTTCCTTTACG |
